# Alternatives Integrating Omics Approaches for the Advancement of Human Skin Models: A Focus on Metagenomics, Metatranscriptomics, and Metaproteomics

**DOI:** 10.3390/microorganisms13081771

**Published:** 2025-07-29

**Authors:** Estibaliz Fernández-Carro, Sophia Letsiou, Stella Tsironi, Dimitrios Chaniotis, Jesús Ciriza, Apostolos Beloukas

**Affiliations:** 1 Tissue Microenvironment (TME) Lab, Aragón Institute of Engineering Research (I3A), University of Zaragoza, C/Mariano Esquillor s/n, 50018 Zaragoza, Spain; estifernandez@unizar.es (E.F.-C.);; 2 Department of Anatomy and Histology, Faculty of Medicine, University of Zaragoza, 50009 Zaragoza, Spain; 3 Department of Biomedical Sciences, University of West Attica, Ag. Spiridonos St. Egaleo, 12243 Athens, Greece; stellatsironi@gmail.com (S.T.); dchaniotis@uniwa.gr (D.C.); 4 Department of Food Science and Technology, University of West Attica, Ag. Spiridonos St. Egaleo, 12243 Athens, Greece; 5 Institute for Health Research Aragón (IIS Aragón), Avda. San Juan Bosco, 13, 50009 Zargoza, Spain; 6 Center of Bioengineering, Biomaterials and Nanomedicine, CIBER-BBN, 28029 Madrid, Spain

**Keywords:** skin microbiome, metagenomics, metatranscriptomics, metaproteomics, human skin models

## Abstract

The human skin microbiota, a complex community of bacterial, fungal, and viral organisms, plays a crucial role in maintaining skin homeostasis and regulating host-pathogen interactions. Dysbiosis within this microbial ecosystem has been implicated in various dermatological conditions, including acne vulgaris, psoriasis, seborrheic dermatitis, and atopic dermatitis. This review, for the first time, provides recent advancements in all four layers of omic technologies—metagenomics, metatranscriptomics, metaproteomics, and metabolomics—offering comprehensive insights into microbial diversity, in the context of functional skin modeling. Thus, this review explores the application of these omic tools to in vitro skin models, providing an integrated framework for understanding the molecular mechanisms underlying skin–microbiota interactions in both healthy and pathological contexts. We highlight the importance of developing advanced in vitro skin models, including the integration of immune components and endothelial cells, to accurately replicate the cutaneous microenvironment. Moreover, we discuss the potential of these models to identify novel therapeutic targets, enabling the design of personalized treatments aimed at restoring microbial balance, reinforcing the skin barrier, and modulating inflammation. As the field progresses, the incorporation of multi-omic approaches into skin-microbiome research will be pivotal in unraveling the complex interactions between host and microbiota, ultimately advancing therapeutic strategies for skin-related diseases.

## 1. Introduction

The skin surface harbors a largely symbiotic community of bacterial, fungal, and viral organisms collectively referred to as the skin microbiota [1]. This community resides on the surface of the epidermis as well as within skin appendages and invaginations, such as hair follicles, sebaceous glands, and sweat glands. Recent findings have also revealed that certain bacteria colonize the dermis [2,3]. This symbiotic and resident microbiota plays a critical role in maintaining skin homeostasis by preventing pathogen colonization, modulating immune responses, regulating epidermal differentiation, and producing nutrients through the breakdown of natural substrates [1,4]. Together, skin cells, microorganisms, and the local microenvironment create a complex ecosystemic across different skin regions. The skin microbiota is highly individualized, resembling a microbial fingerprint unique to each person. However, under certain conditions, disruptions in this equilibrium—termed dysbiosis—may lead to alterations in microbial populations and the development of skin disorders or infections [3].

From birth, the human skin is colonized by millions of microorganisms, with initial composition heavily dependent on the mode of delivery. Newborns delivered via caesarean section harbor microbiota resembling maternal skin, whereas vaginally delivered infants exhibit a microbiota more similar to the vaginal microbiome [5]. While the skin microbiota is initially homogenous across the body during the first three months of life, it becomes region-specific as development progresses, influenced by skin type (moist, dry, sebaceous, or foot), and stabilizes by the first year of life [4,6]. Puberty induces substantial shifts in the microbiota, driven by hormonal changes and sexual maturation. Increased sebum production during puberty promotes the predominance of certain lipophilic bacteria, such as Cutibacterium acnes (formerly Propionibacterium acnes), which metabolizes sebum under controlled conditions. However, when C. acnes overgrows, dysbiosis ensues, contributing to acne vulgaris disease. In adulthood, the skin microbiota stabilizes into distinct communities across different body sites [4,7,8] with four major bacterial phyla dominating: Actinobacteria (51.8%), Firmicutes (24.4%), Proteobacteria (16.5%), and Bacteroidetes (6.3%) [9].

Disruption of microbial homeostasis has been linked to a variety of dermatological disorders, including acne vulgaris, psoriasis, and seborrheic dermatitis. During puberty, the increased sebum levels favor the abundance of Cutibacterium spp. bacteria, exacerbating acne vulgaris. In psoriasis, while the etiology remains unclear, several studies have indicated that an increased abundance of Staphylococcus aureus and Staphylococcus pyogenes, alongside a decrease of C. acnes, may be significant contributors to disease pathogenesis [10,11]. Seborrheic dermatitis is characterized by Malassezia yeasts, which secrete lipases to break down sebaceous lipids, generating metabolites that activate inflammatory pathways responsible for the erythematous, greasy, and flaky symptoms of the disease [12,13].

Several in vitro skin models seeded with microbiota have been developed to simulate various skin pathologies. However, most models focused on one or a few commensal or pathogen microorganisms, analyzing their effects on tissue morphology and barrier function without delving deeply into the underlying molecular mechanisms. Recognizing the limitations of traditional in vitro models in studying the skin microbiome in depth, researchers have increasingly returned to advanced sequencing and omics techniques. These include metagenomics, metatranscriptomics, metaproteomics, and metabolomics [14,15], which are more powerful tools for examining the diversity, function, and activity of microbial communities in both healthy and diseased skin. Furthermore, recent reviews summarize the utility of omics approaches in skin-microbiota research [1,16,17,18]. However, to our knowledge, no recent review has provided a detailed integration of all four major omic layers—metagenomics, metatranscriptomics, metaproteomics, and metabolomics—in the context of functional skin modeling.

## 2. Metagenomics

Today, two of the most used next-generation sequencing (NGS) techniques are 16S rRNA gene sequencing (16S) and whole metagenome sequencing (WMS). Metagenomics involves the genomic analysis of entire microbial communities through DNA extraction and sequencing methodologies [15,19]. The 16S rRNA sequencing, also known as metataxonomic sequencing, employs primers that bind to specific conserved regions of the hypervariable loop within bacterial ribosomal RNA genes, followed by PCR amplification [15,20]. Although this approach primarily targets bacterial genomes, it can also be applied to eukaryotic organisms [15,21]. By targeting conserved fungal-specific ribosomal RNA genes, such as the internal transcribed spacer (ITS1-ITS2), the 18S ribosomal small subunit RNA gene, or the D1/D2 domain of the 26S ribosomal large subunit RNA gene, [15,22]. The utility of amplicon sequencing lies in the availability of comprehensive reference genomic databases that aid in [15,23].

Despite its widespread use, amplicon sequencing has several limitations. The main drawbacks are its reliance on conserved genomic regions, which may limit the resolution of species identification, and the occurrence of PCR amplification artifacts, such as chimeric sequences, which can distort taxonomic assignment and decrease the quality of sequencing reads [15,19,23,24]. Whole Metagenome Sequencing (WMS), a more advances NGS technology, can detect approximately twice as many species as amplicon sequencing at comparable reading depths [15,25]. Unlike amplicon sequencing, WMS does not rely on primers to target specific genes. Instead, all DNA present in the sample, including both host and microbial DNA, is fragmented and sequenced independently [15,20].

The disadvantages of WMS are primarily related to cost and complexity. Amplifying and sequencing the entirety of genomic material in a sample is considerably more expensive and technically demanding than amplicon sequencing. Furthermore, the comprehensive nature of WGS-generated data (host and microbial) requires extensive computational resources and time for data analysis. This complexity is compounded by the need to filter out host DNA, which further increases the computational demands. Additionally, achieving sufficient sequencing depth in skin-microbe studies can be challenging, though advancements in technology continue to address these issues [15,26].

Between the two technologies, WMS is increasingly being favored in skin-microbiome research due to its growing WMS reference databases and its ability to provide a more comprehensive and accurate representation of microbial diversity [15,24]. Both sequencing technologies offer valuable insights. Metagenomics allows for the reliable identification of microbial diversity, assessing species presence and relative abundance. These techniques also provide insight into the functional potential of the microbiome, sequencing microbiome genomes at the species level and, in some cases, at the strain level. This enables the study of prokaryotes, archaea, viruses, bacteriophages, and eukaryotes, while facilitating the functional classification of gene sequences, is allowed. Ultimately, this leads to the discovery of new microbial genes and genomes [15,25].

In the context of human skin-microbiome research, metagenomic applications primarily focus on understanding the bacterial composition and its variability across different individuals and body sites [27]. Furthermore, metagenomics is integral in exploring the relationship between microbial populations and skin diseases, particularly whether the presence of certain bacteria or microbial communities is a cause or consequence of pathologies such as eczema and atopic dermatitis [27,28]. Recent workflows integrating metaproteomics and metagenomics, such as Unipept-based biodiversity profiling, have improved the resolution of microbial community function in skin environments [29].

## 3. Metatranscriptomics

Metatranscriptomic analysis involves the characterization of the transcriptomic profile of microbial communities through the study of RNA molecules [30]. The strength of metatranscriptomics lies in its ability to reveal the functional expression of microbial genes, providing insights into the activity of microorganisms even when cultured in vitro [15,26]. The typical workflow begins with sample collection and the isolation of messenger RNA (mRNA). Once purified, mRNA is reverse transcribed into complementary DNA (cDNA), which is then sequenced in parallel with transgenomic samples [15,31]. The generated sequences, known as RNA-Seq, are subsequently aligned with reference databases for functional analysis.

In the field of human dermal microbiome research, metatranscriptomics is infrequently applied due to its resource-intensive nature and the technical challenges associated with mRNA isolation. One significant obstacle is the potential contamination of microbial RNA with host-derived RNA, which can occur during sample collection. Despite these difficulties, complementing WMS with metatranscriptomics data allows for a more accurate assessment of the gene expression level, providing functional insights that complement genomic information obtained through metagenomic analysis [15,26].

By analyzing the transcriptomic data, researchers can identify active metabolic pathways within microbial communities across various environments, which holds significant potential for biomedical advancements [15,26]. Metatranscriptomics not only enhances the understanding of which genes reported in metagenomic studies are actively transcribed but also quantifies the degree of expression, providing a detailed view of gene functionality [23,27]. This functional information enables the identification of metabolic pathways that are active in bacterial populations, correlating their activity with environmental conditions and potential therapeutic targets [23,28]. However, challenges in metatranscriptomic analysis of skin samples due to low biomass and host RNA contamination remain significant, as highlighted in recent multi-omics reviews [18].

Thus, metatranscriptomics offers a deeper understanding of microbial communities by focusing on transcriptionally active populations, rather than solely identifying the genetic content of these communities. This approach allows researchers to explore gene expression dynamics in response to environmental changes, revealing new insights into microbial functionality and interaction [26,31,32]. To this, *metaproteomics* can also contribute.

## 4. Metaproteomics

Metaproteomics is defined as the large-scale study of the complete protein content produced by the environmental microflora at a given time [15,33]. Identifying and quantifying the proteomic landscape enables researchers to analyze the molecular components supporting microbial ecosystem survival. Typically, protein identification and quantification of proteins are achieved using shotgun proteomics, where peptides are enzymatically cleaved and subsequently analyzed by liquid chromatography-mass spectrometry [15,33,34]. The resulting data provide insights into the amino-acid sequences, protein abundances, and post-translational modifications, such as phosphorylation. These sequences are then compared against online reference databases to precisely identify the proteins present [15,35].

One of the key outcomes of metaproteomic analysis is understanding which microorganisms are actively contributing to the ecosystem’s function by examining their protein expression [36,37]. This functionality is characterized by two main parameters: the association of proteins with functional units and their relative abundance, which serves as an indicator of their metabolic activity. The analysis of proteins secreted or released by microbial cells provides insights into how these cells interact with each other and their environment [37,38,39]. Moreover, the identified peptides can be traced back to the organisms responsible for producing them, enabling a detailed molecular characterization of their phenotype [37,40,41,42].

Despite its potential, metaproteomics has been underutilized in skin-microbiome research, particularly compared to its widespread application in gut microbiome studies [31,37,38]. Several factors, including limited sequencing depth and high cost of proteomic analysis, may account for this discrepancy. However, advancements in biotechnology are making metaproteomics applications in skin research increasingly feasible. Metaproteomic techniques offer a range of capabilities, such as monitoring functional genes and metabolic pathways, tracking protein expression under stress conditions, and aiding in the discovery of novel functional genes. All these capabilities are invaluable for unraveling the role of microbes in the onset and progression of skin diseases [15]. As biotechnology continues to evolve, integrating metaproteomics into skin-microbiome research will provide deeper insights into the functional dynamics of microbial communities, ultimately contributing to a better understanding and treatment of skin disorders. In addition, metabolomics, as part of the integrated omics approach, can also aid in the functional dynamics of skin microbiota.

## 5. Metabolomics

The final category of the four “omics” disciplines considered here is metabolomics, part of the integrated omics approach, which involves the identification and quantification of the complete set of metabolites present in a sample. Similar to metaproteomics, metabolites are identified and quantified using advanced analytical techniques, including liquid and gas chromatography, mass spectrometry, and nuclear magnetic resonance. The quality of the obtained results depends significantly on the purity and preparation of the collected samples. As with the genomic sequencing, metabolomic data are compared with known spectral databases to elucidate the identity and concentration of metabolites [15,43].

While most current metabolomics research focuses on the gut microbiome, studies in the field of the skin microbiome are gradually emerging. For instance, an important study by Kuehne et al. demonstrated that aging induces only minor metabolomic and transcriptional changes in the skin [15,43,44]. One key area where metabolomics has shown promise is lipidomics, particularly in the study of psoriasis, where it has helped elucidate the role of lipid metabolism in disease pathogenesis [15,45]. This methodology allows researchers to note the identity and quantify metabolites within a sample, but also to understand their functional roles in metabolic pathways. Recent LC–LC-MS-based metabolomics profiling of psoriatic lesions has identified key lipid and amino-acid metabolites linked to inflammation and disease progression [46].

Despite the economic challenges associated with metabolomic techniques, their use enables researchers to examine complex microbial pathways, such as bacterial communication via signaling molecules [15,43]. When combined with WMS, metabolomics provides a comprehensive framework for reconstructing the intricate complex networks of microbial communities [15]. This integration of multiple “omics” approaches opens new avenues for understanding the functional dynamics of microbial ecosystems and their roles in both health and disease.

## 6. Building In Vitro Skin Models

Much of the current research on the skin microbiome still relies on amplicon sequencing, due to its lower cost and reduced labor requirements. However, advances in biotechnology have made NGS methods—particularly whole metagenome sequencing (WMS)—increasingly valuable. WMS offers higher resolution, allowing for strain-level identification and the prediction of gene functions and metabolic capabilities, making it a powerful tool in microbiome research. This is of particular importance because certain strains within the *Staphylococcus* spp. genus, for instance, is associated with either healthy skin or pathological skin conditions [42]. However, many researchers studying the skin microbiome have yet to incorporate other “omics” techniques into their research. A recent in-depth review emphasizes emerging concepts and gaps, with implications of skin microbiota into functional models of skin–microbe interactions [1]. Another recent narrative review addresses how multi-omics tools (genomics, epigenomics, proteomics, metabolomics, and microbiomics) can refine diagnosis and therapy in immune-mediated skin diseases [17].

The combination of metagenomics with metatranscriptomics, metaproteomics, and/or metabolomics offers a more comprehensive and detailed understanding of host-microbiota interactions in both healthy and diseased states. As research on the skin-microbiome advances, the focus must move beyond identifying microbial species to exploring their functional roles—specifically, what these microbes actually do. Investigating the products they produce, how these are synthesized, and their effects on skin health will offer deeper insight into skin disorders. As sequencing depth requirements and technical complexity diminish, the incorporation of additional “omics” approaches into skin-microbiome research is inevitable [43]. In addition, recent advancements in microfluidic skin-on-a-chip platforms have enabled integration of immune and vascular systems, mimicking more physiologically relevant environments for host-microbe interactions [47,48,49,50]. These models support complex inflammatory testing and microbiome modulation studies.

By employing methods such as metatranscriptomics and metaproteomics, researchers can develop more advanced in vitro skin models that incorporate multiple layers and cellular components of the skin microenvironment, including immune system elements and skin microbiota under both healthy and diseased conditions. These approaches allow for detailed analysis of skin–microbiome interactions, offering valuable insights into skin biology and pathology. Ultimately, such studies may facilitate the development of targeted therapies aimed at improving skin health and preventing or treating skin diseases by elucidating the underlying biological mechanisms.

In recent years, dermatological research has made notable progress in developing in vitro skin models with integrated microbiota. However, further research is needed to fully understand the specific microbiota present and their functional roles. For example, in a full-thickness dry skin model, *Staphylococcus epidermidis*, *C. acnes*, and *Malassezia furfur* as commensal microorganisms, and *S. aureus*, as an opportunistic bacterium, were introduced individually as representatives of the skin microbiota. Their findings revealed that colonization by *S. aureus* led to a faster deterioration of the skin barrier compared to colonization by commensal microorganisms [51]. If microorganisms had been introduced simultaneously, metagenomics would have facilitated their identification and quantification, illustrating the influence of specific bacteria populations. Furthermore, the use of metatranscriptomics, metaproteomics, and metabolomics would have helped to elucidate how skin microenvironment factors such as pH or the presence of other pathogenic organisms could affect gene expression, protein expression, and metabolite production, providing insights into treatments aimed at enhancing protective metabolic pathways and metabolite production.

To investigate acne vulgaris, researchers applied a combination of peroxidized squalene and a population of *C. acnes* to the surface of a 3D in vitro skin model to simulate sebum alteration and microbial invasion. This model successfully reproduced several characteristics of acneic skin, including hyperkeratinization of the *stratum corneum*, activation of toll-like receptor-2 (TLR-2) by *C. acne* presence involved in immune activation, and elevated secretion of inflammatory cytokines by keratinocytes [52,53]. These experiments used either commercially sourced *C. acnes* strains or bacteria isolated from acne patients. In this context, metagenomics plays a critical role, as certain *C. acne* strains are implicated in acne vulgaris development, while others are associated with healthy skin [54]. Additional omics tools could further elucidate the etiology of acne lesions, addressing the debate over whether inflammatory processes or compositional modifications in sebum and hyperkeratinisation are the primary initiators [44,47]. For example, metatranscriptomics could analyze gene expression patterns in *C. acnes* from acne-affected versus healthy skin, identifying genes that are activated during colonization and contribute to inflammation and lesion formation.

Atopic Dermatitis (AD), a common inflammatory skin disorder, is influenced by immune dysregulation, environmental factors, and microbiota dysbiosis, caused by an overgrowth of *S. aureus*, with the severity of AD often linked to increases in this bacterium [55,56,57]. In 2D keratinocyte models, interferon-λ1 (IFN-λ1) has been shown to inhibit *S. aureus* colonization, restore skin barrier function, and modulate skin degradation [58]. Omic tools have provided comprehensive molecular insights into the disease seen in AD, including the role of INF-λ1, and have identified potential therapeutic targets. A systematic review covering genomics, transcriptomics, proteomics, metabolomics, and microbiomics applied to psoriasis and atopic dermatitis discusses biomarkers, pathogenic mechanisms, and strategies for integrated, systems-biology approaches [18]. In a recently published study, researchers used hiPSC-derived 3D skin organoids to simulate *S. aureus* colonization in an AD model. As in the previous study, they found that *S. aureus* altered the skin barrier and increased the production of inflammatory cytokines in the epidermis and dermis. However, when the model was pre-treated with commensal *C. acne* bacteria two days prior to *S. aureus* colonization, no barrier damage was observed, indicating the protective effect of commensal bacteria [59]. Metagenomics could have facilitated the identification of alterations that promote *S. aureus* colonization, potentially leading to personalized therapeutic strategies aimed at activating commensal bacteria competing with *S. aureus* to restore microbial balance. Moreover, metatranscriptomics and metaproteomics could elucidate the activated or blocked metabolic pathways, pro-inflammatory proteins, or toxins generated by *S. aureus*, identifying potential therapeutic targets. In seborrheic dermatitis (SD), although its precise etiology remains unclear, evidence suggests a correlation between the prevalence of *Malassezia* spp. fungi and disease severity, [60,61] particularly with *M. globosa* and *M.stricta* strains. This inflammatory and scaly skin condition affects sebaceous gland-rich areas such as the scalp and is characterized by dandruff [62]. A 2D keratinocyte monolayer model was used to explore the epidermis’ defense mechanisms against colonization by pathogenic *M. globosa* or *M. restricta* strains. Compared to *M. furfur*, these strains exhibited cytotoxicity in keratinocytes, modulating the inflammatory response by blocking TLR-2, which plays a role in the host defense against fungal infections [55].

In another study, a 3D epidermal model demonstrated that *C. acnes* is beneficial for scalp health, while *M. restricta* induces dandruff. Upon exposure, the epidermal barriers colonized by *M. restricta* exhibited significant damage, including reduced transepithelial electrical resistance (TEER) and altered expression of the epidermal markers, such as cytokeratin 10, cytokeratin 14, involucrin, and loricrin. In contrast, samples colonized by *C. acne* showed no such damage. The damage observed in the model co-colonized by both microorganisms was less severe, indicating that *C. acnes* can protect the skin against pathogens, maintaining host-microbiota homeostasis [63,64]. Metabolomics could have been employed to analyze the metabolites produced by *M. restricta* and their impact on the skin microenvironment, revealing their role in inflammation and disease progression. Furthermore, metabolomics could have elucidated the metabolic pathways used by the yeast to metabolize skin lipids, essential for its survival and pathogenesis, potentially identifying inhibitory or modulatory targets to restore skin homeostasis.

Considering the preceding models, an advanced in vitro skin model incorporating omic data would offer valuable insights into the skin-microbiota population. The development of such a model should follow a series of well-defined steps.

**Step 1: Establishment of an in vitro skin model without microbiota**

Before introducing the microbial population, it is essential to establish a robust in vitro skin model. Existing models can be either two-dimensional (2D) or three-dimensional (3D). While 2D models are relatively simple and easy to use, they often result in altered cellular morphology, proliferation capacity, differentiation potential, and gene expression. In contrast, 3D models accurately replicate the structural complexity of human skin, though they are more expensive and technically challenging to establish [65]. A 3D skin model should comprise both dermis and a differentiated epidermis, including immune and endothelial cells to simulate the immune system and blood vessels, respectively.

**Figure 1:**
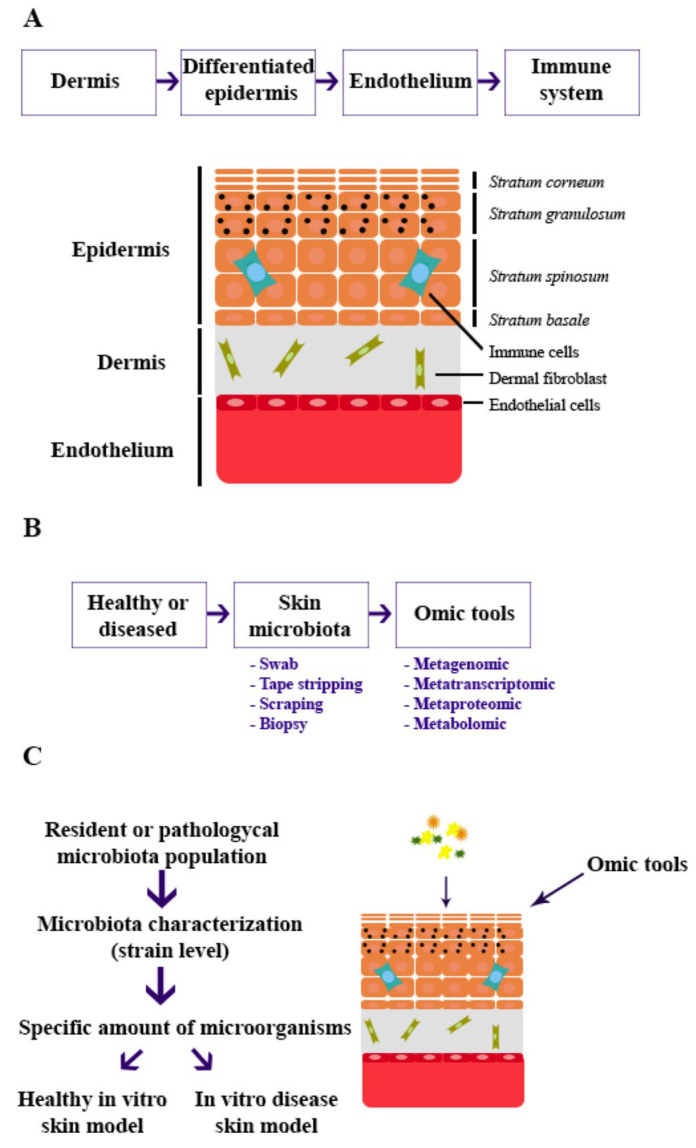
Timeline of (**A**) establishment of in vitro skin model, (**B**) sample collection from donors and data extraction, and (**C**) application of skin microbiota in the skin model.

To achieve this, dermal fibroblasts must first be embedded in a scaffold composed of natural or synthetic materials, such as type I collagen, dermal extracellular matrix, or polyethersulfone (PES) [66,67], to mimic the dermis layer. Keratinocytes are then seeded onto the scaffold to form the epidermis. It is critical to ensure that the keratinocytes are fully differentiated into the four epidermal layers—stratum basal, stratum spinosum, stratum granulosum, and stratum corneum—to accurately represent the skin’s protective barrier, a key component in host-microbiota interactions. For this purpose, the use of primary keratinocytes isolated from skin donors is recommended [68]. Additionally, microvascular endothelial cells, simulating blood vessels, and immune cells should be incorporated to explore immune cells-skin-microbiota interactions within the model (Figure 1A) [69].

**Step 2: Sample collection and omic data collection integration**

The microbial population to be incorporated into the in vitro skin model should be determined based on omic data collected from healthy and/or diseased skin donors. The model must consider the specific skin site to be studied, as the physiology of each skin location creates a unique and specific niche for the microbiota. Additionally, researchers must determine whether the focus is on healthy microbiota or dysbiosis, and the sample collection method should be tailored accordingly. Commonly used techniques include swab, tape stripping, scraping, and skin biopsy, the most common techniques [62,63,64]. (Table 1).

After collection, samples should be stored at −80 °C or in stabilization tubes to preserve their integrity until processing by omic techniques [70]. Metagenomic analysis will provide detailed information on the exact composition of the microbiota at the strain level in both healthy and pathological skin samples, allowing researchers to replicate the appropriate microbiota population in the in vitro model. This level of precision will ensure that the skin microenvironment is accurately represented, allowing for more meaningful insights into microbial activity and host-microbiota interactions (Figure 1B).

**Step 3: Addition of microbiota in the in vitro skin model**

The microbial population introduced into the in vitro model should be selected based on the research focus, representing healthy or pathological skin conditions. To obtain the microbial communities of interest, microorganisms can be isolated directly from donor samples, either healthy or pathological. However, this method is complex, subject to donors’ variability, and carries a high risk of contamination [70,71,72]. An alternative, microorganisms can be sourced from commercially available pure bacterial biobanks.

Quantified bacterial populations, previously characterized through metagenomic analysis, can then be introduced into the 3D skin model. The evolution of the microbial community can be monitored by molecular techniques such as real-time qPCR, 16S rDNA PCR, multiplex PCR, or microarrays [73]. This model can be applied in various fields, including physiological studies and pharmacological testing, allowing for the assessment of microbial dynamics before and after treatment exposure. Sampling techniques such as swabbing can be used, followed by the application of metagenomics, metatranscriptomics, metaproteomics, or metabolomics to analyze the molecular interactions within the model (Figure 1C).

**Step 4: Model validation**

All in vitro models must undergo validation to ensure their accuracy and applicability. For 3D skin models, it is recommended to follow the recommendations set forth in the *New Developments in Skin and Epidermal Equivalent Models at the 2019 Barrier Function of Mammalian Skin Gordon Research Conference*. This conference established key validation parameters for evaluating the quality and suitability of skin models in barrier function research. In terms of quality, essential parameters include the morphology and epidermal stratification, which are assessed through histological analysis and immunohistochemistry/immunofluorescence, respectively. For studies focused on skin barrier function, it is recommended to evaluate the model’s permeability, both from inside to outside and outside to inside, to ensure accurate replication of barrier properties. In this consensus, in vitro 3D skin models incorporating microbial integration were also considered. It was agreed that the viability of microorganisms should be examined before and after co-culture using techniques such as DAPI, FISH, or fluorescence [74]. Incorporating omics tools into the validation process is also recommended, as they provide a deeper understanding of the model’s functionality at the molecular level.

## 7. Conclusions

This work focuses on in vitro skin models as platforms to integrate omics data and proposes a clear roadmap for developing validated models that incorporate microbial communities. Specifically, it highlights the development of advanced in vitro skin models, coupled with the integration of omic tools, facilitating a comprehensive understanding of the roles played by skin commensal microbiota. Thus, these skin models will provide deeper insights into dermatological pathologies associated with microbiota dysbiosis, while also identifying novel therapeutic targets. The ability to study microbial interactions in these models will enable the development of treatments that restore microbial balance, reinforce the skin barrier, and modulate inflammation. Ultimately, this will pave the way for more effective, personalized therapies, tailored to address the unique microbial dynamics of individual patients.

## Figures and Tables

**Table 1:** Comparative table of skin-microbiota sampling techniques.

	Collecting Area	Advantages	Disadvantages
Swab	Surface and epidermal layers	Easy No invasive Commercially available Standardized	Often contain small bacterial yields
Tape stripping	Deep epidermal layers	Easy Commercially available More cultivable bacteria collected	Different adhesives Less efficient in oily, wet, or undulating skin
Scraping	Deep epidermal layers	Easy	Invasive Mechanical scraping damages the skin. DNA contamination (host DNA)
Biopsy	Epidermis and dermis	Most representative skin-microbiota detection	Invasive DNA contamination (host DNA)
